# Plasma lipidome mediates the causal effect of chronic hepatitis B on cirrhosis: A Mendelian randomization study

**DOI:** 10.1097/MD.0000000000048252

**Published:** 2026-04-10

**Authors:** Biao Xiao, Qixiang Song, Zhenggang Wang, Jie Huang, Ying Liu, Boqi Li, Qinghai Zeng, Yelin Deng, Ewelina Krol, Ricardo Khouri, Erik De Clercq, Min Zhang, Guangdi Li

**Affiliations:** a Hunan Provincial Key Laboratory of Clinical Epidemiology, Xiangya School of Public Health, Central South University, Changsha, China; b Department of Dermatology, The Third Xiangya Hospital, Central South University, Changsha, China; c Department of Civil and Environmental Engineering, Soochow University, Suzhou, China; d Laboratory of Recombinant Vaccines, Intercollegiate Faculty of Biotechnology, University of Gdansk and Medical University of Gdansk, Gdansk, Poland; e Laboratory of Precision Medicine and Public Health, Gonçalo Moniz Institute, Oswaldo Cruz Foundation, Salvador, Brazil; f Department of Microbiology, Immunology and Transplantation, Rega Institute for Medical Research, KU Leuven, Leuven, Belgium; g Institute of Hepatology and Department of Infectious Diseases, The Second Xiangya Hospital, Central South University, Changsha, China.

**Keywords:** chronic hepatitis B, cirrhosis, mediation, Mendelian randomization

## Abstract

This study aimed to investigate the role of the plasma lipid species in the progression from chronic hepatitis B (CHB) infection to cirrhosis. We conducted 2-sample Mendelian randomization (MR) and genome-wide mediation analyses to explore the causal relationship between CHB and cirrhosis, focusing on lipid species as potential mediators. The inverse variance weighted method was used as the primary analytical approach, supported by complementary estimators. Sensitivity analyses – including Cochran’s *Q* test, MR-Egger intercept test, of Mendelian randomization pleiotropy residual sum and outlier global test, and leave-one-out analysis – were performed to assess robustness, heterogeneity, and horizontal pleiotropy. We found a significant positive association between CHB and cirrhosis (odds ratio = 1.206; 95% CI: 1.080 to 1.346; *P* = .001). Among 179 common lipid species analyzed, genetic predispositions for 29 were significantly associated with CHB and 4 with cirrhosis. Mediation analysis identified 2 lipid species – ceramide (d40:2) and phosphatidylcholine (18:2_20:4) – as potential mediators in the causal pathway from CHB to cirrhosis. Ceramide (d40:2) mediated 11.65% of the effect (mediation effect: 0.022; 95% CI: 0.002 to 0.049; *P* = .037), while phosphatidylcholine (18:2_20:4) mediated 6.62% (mediation effect: 0.012; 95% CI: −0.0003 to 0.030; *P* = .072). Overall, this study provides genetic evidence supporting the causal role of CHB in the development of cirrhosis and highlights ceramide (d40:2) and phosphatidylcholine (18:2_20:4) as key mediators in this process.

## 1. Introduction

Cirrhosis is a global public health problem, with its mortality rate steadily increasing in recent years. In 2019, cirrhosis was associated with 2.4% of global deaths.^[1]^ Chronic hepatitis B (CHB) virus infection is the main risk factor for cirrhosis. Globally, CHB affects about 296 million people and is the leading cause of cirrhosis and liver cancer, with up to 42% of cirrhosis patients attributable to HBV infection.^[2,3]^ HBV-related cirrhosis resulted in approximately 3,31,000 deaths in 2019.^[4]^ During the long-term HBV infection, the liver suffers from continuous injury with an increased risk of cirrhosis.^[5]^ Managing CHB will reduce the risk of developing cirrhosis, and a deeper understanding of the progression from CHB to cirrhosis is critical for improving prevention and treatment strategies.

The lipidome, a complex network of diverse lipid species, plays a critical role in many physiological and pathological processes.^[6]^ Dysregulated lipid metabolism contributes significantly to the pathogenesis of metabolic liver disorders such as cirrhosis.^[7,8]^ Specific lipids, including ceramide, lysophosphatidylcholine, and phosphatidylcholine, have been proposed as clinical biomarkers and noninvasive diagnostic indicators of liver disease.^[9-11]^ Dynamic changes in the abundance and composition of these lipids are strongly associated with the pathogenic mechanisms of CHB.^[12-14]^ Moreover, the importance of the lipidome in liver disease progression has been reported in the literature.^[6]^ However, it remains unclear whether – and by what mechanisms – the lipidome mediates the critical transition from CHB to cirrhosis, a pathological cascade with major implications for disease severity and patient prognosis.

Recently, Mendelian randomization (MR) has become a popular epidemiological method that addresses potential causal relationships between exposures and outcomes by using genetic variations as instrumental variables and large-scale data from genome-wide association studies (GWASs).^[15]^ MR provides a robust approach to addressing the limitations of observational studies, as genetic variants are randomly allocated at conception and are not influenced by measurement error, confounding, or reverse causality.^[16]^ In addition, mediation analysis dissects the overall causal effect of an exposure on an outcome by examining the indirect effect of the exposure on the outcome through a mediating factor.^[17]^ Here, this study conducted MR analysis using publicly available GWAS data to explore whether the lipid species act as mediators in the progression from CHB to cirrhosis. Specific types of lipid species and their cellular pathways are also investigated.

## 2. Materials and methods

### 2.1. Study design

**Figure 1.**
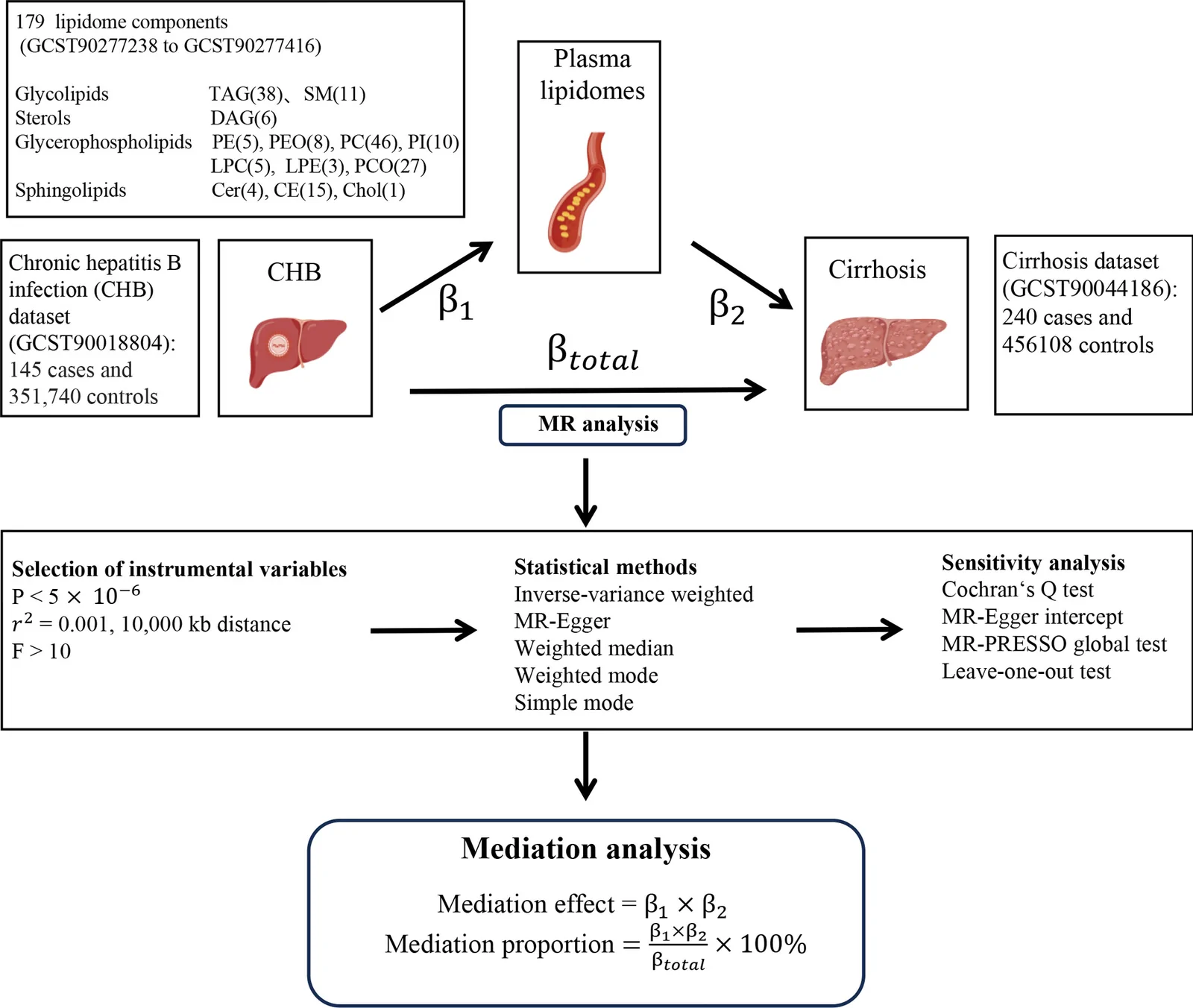
The overview of our study design. CHB = chronic hepatitis B, MR = Mendelian randomization, MR-PRESSO = Mendelian randomization pleiotropy residual sum and outlier.

Our study design is visualized in Figure 1. Based on 3 principles of MR,^[18,19]^ we defined single-nucleotide polymorphisms (SNPs) as the instrumental variables, which are closely associated with the exposure factor, but they are independent of confounding factors. Moreover, the instrumental variables are not directly related to the outcome, but only influence the outcome through the exposure factor. Our analyses mainly consisted of 3 steps. In step 1, we analyzed the causal effect of CHB (exposure factor) on cirrhosis (outcome factor). In step 2, we evaluated the associations of 179 lipid species with CHB (exposure factor) and cirrhosis (outcome factor). In step 3, we conducted the mediation analysis of 179 lipid species (mediator factor) in the pathway from CHB to cirrhosis. We used genetic instrumental variables to represent the heritability of these lipids and diseases, which are derived from comprehensive GWAS of the corresponding traits.

### 2.2. Data sources

Based on the NHGRI-EBI GWAS Catalog database (https://www.ebi.ac.uk/gwas/), we extracted 3 GWAS datasets: the GWAS data (catalog ID: GCST90018804) from individuals with CHB (N = 145) in the case group and individuals without CHB (N = 3,51,740) in the control group.^[20]^ The GWAS data (GCST90044186) from individuals with cirrhosis (N = 240) and individuals without cirrhosis (N = 4,56,108).^[21]^ The GWAS data (GCST90277238 to GCST9027741) from Finnish individuals (N = 7174) in the GeneRISK cohort.^[22]^ This dataset contains 179 lipid species belonging to 4 major categories: glycolipids, glycerophospholipids, sphingolipids, and sterols.

### 2.3. Selection of instrumental variables

SNPs were selected as instrumental variables, given the threshold of *P* < 5 × 10^−6^ according to a previous publication.^[23]^ Due to palindromic SNPs, allelic frequency information was used to identify the forward-strand alleles.^[24]^ If multiple SNPs were located within a genomic window of 10,000 nucleotides, only 1 SNP was selected to ensure the independence between different instrumental variables. A threshold of (*r*^2^ = 0.001) was applied to reduce the impacts of linkage disequilibrium, which were estimated using the European samples from the 1000 Genomes Project.^[25]^ For each SNP, an *F*-statistic value was calculated to evaluate the statistical strength of the association between each SNP and the exposure.


$$\begin{array}{l} F=\left(\frac{R^{2}}{1-R^{2}}\right)\times \left(\frac{N-k-1}{k}\right) \end{array}$$

where N indicates the sample size of GWAS, *k* is the number of paired samples, and *R*^2^ is the cumulative explained variance of the selected instrument variables on exposure. *R*^2^ was calculated as $R^{2}=2\times MAF\times \left(1-MAF\right)\times \beta^{2}$, where the parameter MAF is the effect allele frequency and β is the effect estimate of genetic variants in the exposure GWAS.^[26,27]^ SNPs with *F*-statistic values ≥ 10 were considered as robust instrumental variables,^[28]^ while those SNPs with *F*-statistic values < 10 were excluded to avoid weak-instrument bias.^[29]^

### 2.4. Two-sample Mendelian randomization design with mediation analysis

We conducted 2-sample MR based on standard protocols in literature.^[30]^ Specifically, we used inverse variance weighted (IVW) as the main assessment tool, allowing for unbiased causal analysis without pleiotropy.^[31]^ Other methods, including MR-Egger, weighted median, simple mode, and weighted mode, were used as supplements.^[31]^ For sensitivity analyses, the MR-Egger intercept was used to assess horizontal pleiotropy, with *P* ≥ .05 indicating no significant pleiotropy to meet the exclusivity assumption.^[32]^ The approach of Mendelian randomization pleiotropy residual sum and outlier was used to identify potential pleiotropic effects and to remove outlier SNPs.^[33]^ Cochran’s *Q* was used to assess heterogeneity, with *P* ≥ .05 indicating no significant heterogeneity.^[34]^ Leave-one-out sensitivity analysis was also used to assess the robustness of 2-sample MR.^[35]^ The open-source packages of TwoSampleMR and of Mendelian randomization pleiotropy residual sum and outlier in the open-source software of R (version 4.3.3) were used for the analysis of 2-sample MR.

To investigate whether a causal effect of CHB on cirrhosis is mediated via a specific pathway involving lipid species, we conducted a 2-step MR design with mediation analysis.^[10]^ The mediation effect is represented by the product of the causal relationship (denoted as $\beta_{1}$) between CHB and a lipid species, and the causal relationship (denoted as $\beta_{2}$) between a lipid species and cirrhosis.^[36]^ The total effect is represented by the causal relationship between CHB and cirrhosis (denoted as $\beta_{total}$). The proportion of the mediation effect is calculated as follows:


$$\begin{array}{l} Mediation\;proportion=\frac{\beta_{1}\times \beta_{2}}{\beta_{total}}\times 100\% \end{array}$$

The mediation proportion above measures the contribution of the mediation effect to the total effect^[36]^

## 3. Results

### 3.1. The causal relationship between CHB and cirrhosis

**Figure 2.**
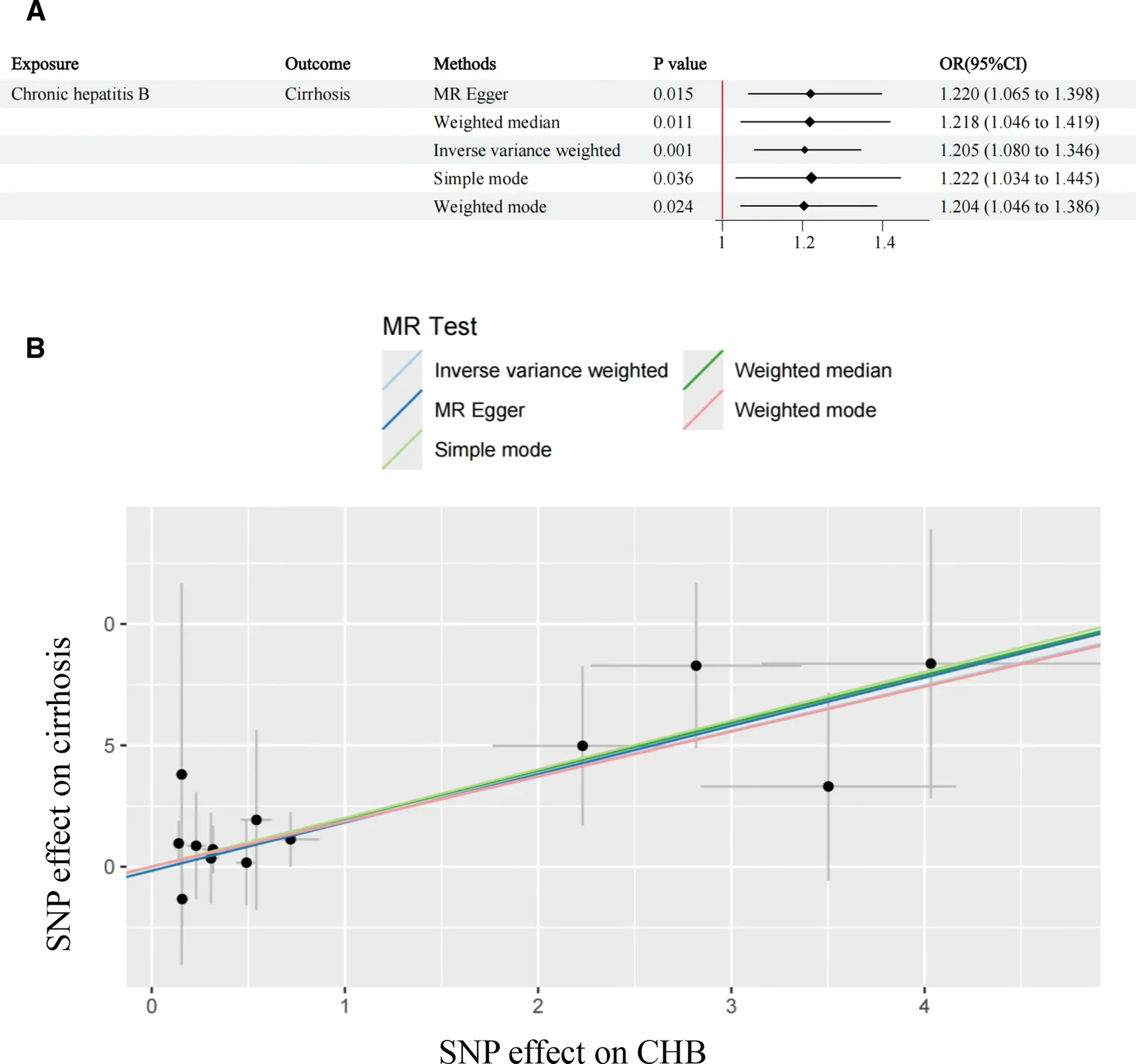
Mendelian randomization analyses confirm the causal effect of CHB on cirrhosis. (A) Forest plot of odds ratios to measure the effect of CHB on cirrhosis using 5 models from 2-sample Mendelian randomization analyses. (B) SNP effects that are consistently reported between CHB and cirrhosis by 5 methods. (C) Leave-one-out sensitivity analysis. CI = confidence interval, CHB = chronic hepatitis B, MR = Mendelian randomization, OR = odds ratio, SNP = single-nucleotide polymorphisms.

After a series of selection steps, a total of 19 instrumental variables were associated with CHB. All *F*-values for inclusion of SNPs > 10 (Table S1, Supplemental Digital Content, https://links.lww.com/MD/R639). The MR analysis revealed significant positive effects of CHB-associated genetic predisposition on the risk of cirrhosis (odds ratio [OR] = 1.206, 95% CI: 1.080 to 1.346, *P* = .001; Fig. 2A). As shown in Figure 2B, this finding was consistently confirmed by using 4 sensitivity methods, including the MR-Egger method (OR = 1.220, 95% CI: 1.065–1.398, *P* = .015), the weighted median method (OR = 1.218, 95% CI: 1.046–1.419, *P* = .011), the simple mode (OR = 1.222, 95% CI: 1.034–1.445, *P* = .037), and the weighted mode (OR = 1.204, 95% CI: 1.046–1.386, *P* = .024; Table S2, Supplemental Digital Content, https://links.lww.com/MD/R639). The robustness of the above findings was further validated by using sensitivity analyses (Tables S3–S6, Supplemental Digital Content, https://links.lww.com/MD/R639).

### 3.2. The causal relationship between CHB and lipid species

**Figure 3.**
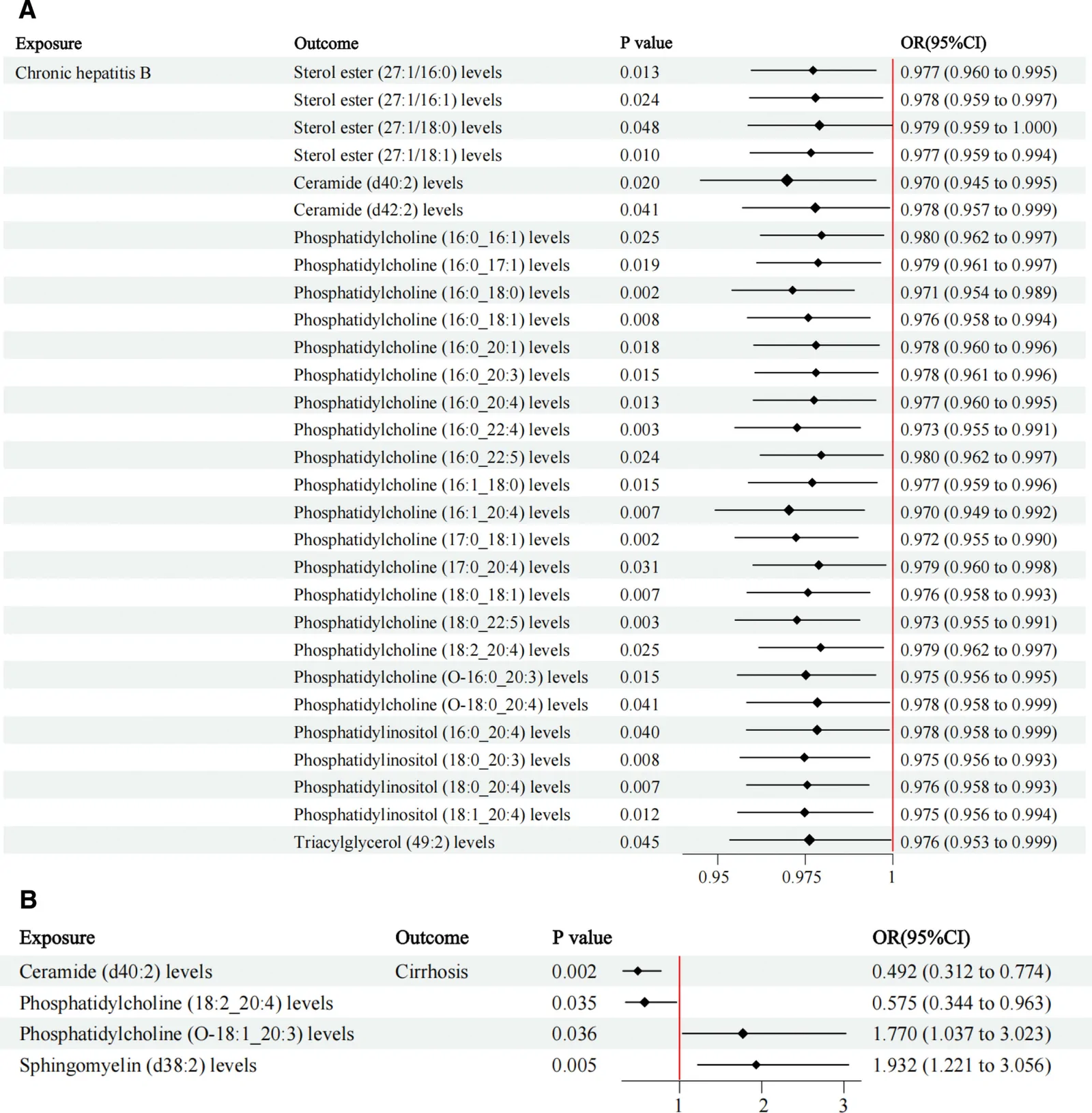
Identification of lipid species associated with either CHB or cirrhosis. (A) The OR values of 29 lipid species with CHB using the IVW method (*P*_IVW_ < .05). (B) The OR values of 4 lipid species with cirrhosis using the IVW method (*P*_IVW_ < .05). CHB = chronic hepatitis B, IVW = inverse variance weighted, OR =odds ratio.

In our investigation on the MR analysis of CHB on plasma lipid species, we observed causal associations of CHB with 29 lipid species (*P*_IVW_ < .05, Fig. 3A). These lipid species can be classified into 4 classes, including glycolipids (N = 1), sphingolipids (N = 2), sterols (N = 4), and glycerophospholipids (N = 22). All these lipid species were negatively correlated with CHB. The negative associations were consistently confirmed by 4 methods, such as the MR-Egger method, the weighted median method, the simple mode, and the weighted mode (Table S7, Supplemental Digital Content, https://links.lww.com/MD/R639). The robustness of the above findings was confirmed using sensitivity analyses (Tables S3–S6, Supplemental Digital Content, https://links.lww.com/MD/R639).

### 3.3. The causal relationship between lipid species and cirrhosis

A total of 1803 SNPs derived from 179 lipid species were employed as instrumental variables. Furthermore, all F statistics have values >10 (Table S8, Supplemental Digital Content, https://links.lww.com/MD/R639). Among 179 lipid species, 4 lipid species had a significant association with the risk of cirrhosis (*P*_IVW_ < .05). As shown in Figure 3B, associations were observed among these 4 lipid species: ceramide(d40:2; OR = 0.492, 95% CI: 0.312–0.774, *P* = .002), phosphatidylcholine (18:2_20:4; OR = 0.575, 95% CI: 0.344–0.963, *P* = .035), phosphatidylcholine (18:1_20:3; OR = 1.770, 95% CI: 1.037–3.023, *P* = .036), sphingomyelin (d38:2; OR = 1.932, 95% CI: 1.221–3.057, *P* = .005). The other 4 methods (the MR-Egger method, the weighted median method, the simple mode, and the weighted mode) showed consistent effect estimates with the IVW estimation, demonstrating the consistency of the MR results (Table S9, Supplemental Digital Content, https://links.lww.com/MD/R639). The robustness of the above findings was further confirmed using sensitivity analyses (Tables S3–S6, Supplemental Digital Content, https://links.lww.com/MD/R639).

### 3.4. Lipid species act as mediators in the causal pathway from CHB to cirrhosis

We performed IVW 2-sample MR analyses to evaluate whether lipid metabolites serve as mediators in the causal pathway from CHB to cirrhosis. First, we estimated the total causal effect (β = 0.188) of CHB on cirrhosis, suggesting that CHB may act as a risk factor in causing cirrhosis. Next, we screened all 179 lipid species and identified 2 lipid species as key mediators: ceramide (d40:2) and phosphatidylcholine (18:2_20:4; Fig. 4). Ceramide (d40:2) exhibited a mediation effect of 0.022 (95% CI: 0.002–0.049), accounting for 11.65% of the total causal effect. Phosphatidylcholine (18:2_20:4) exerted a mediation effect of 0.012 (95% CI: −0.0003–0.030), explaining 6.62% of the total causal effect, but this effect did not reach statistical significance (Fig. 5, Table S10, Supplemental Digital Content, https://links.lww.com/MD/R639) These results highlight the important role of specific lipid species in CHB progression and provide mechanistic insights with potential implications for targeted prevention and therapeutic strategies.

**Figure 4.**
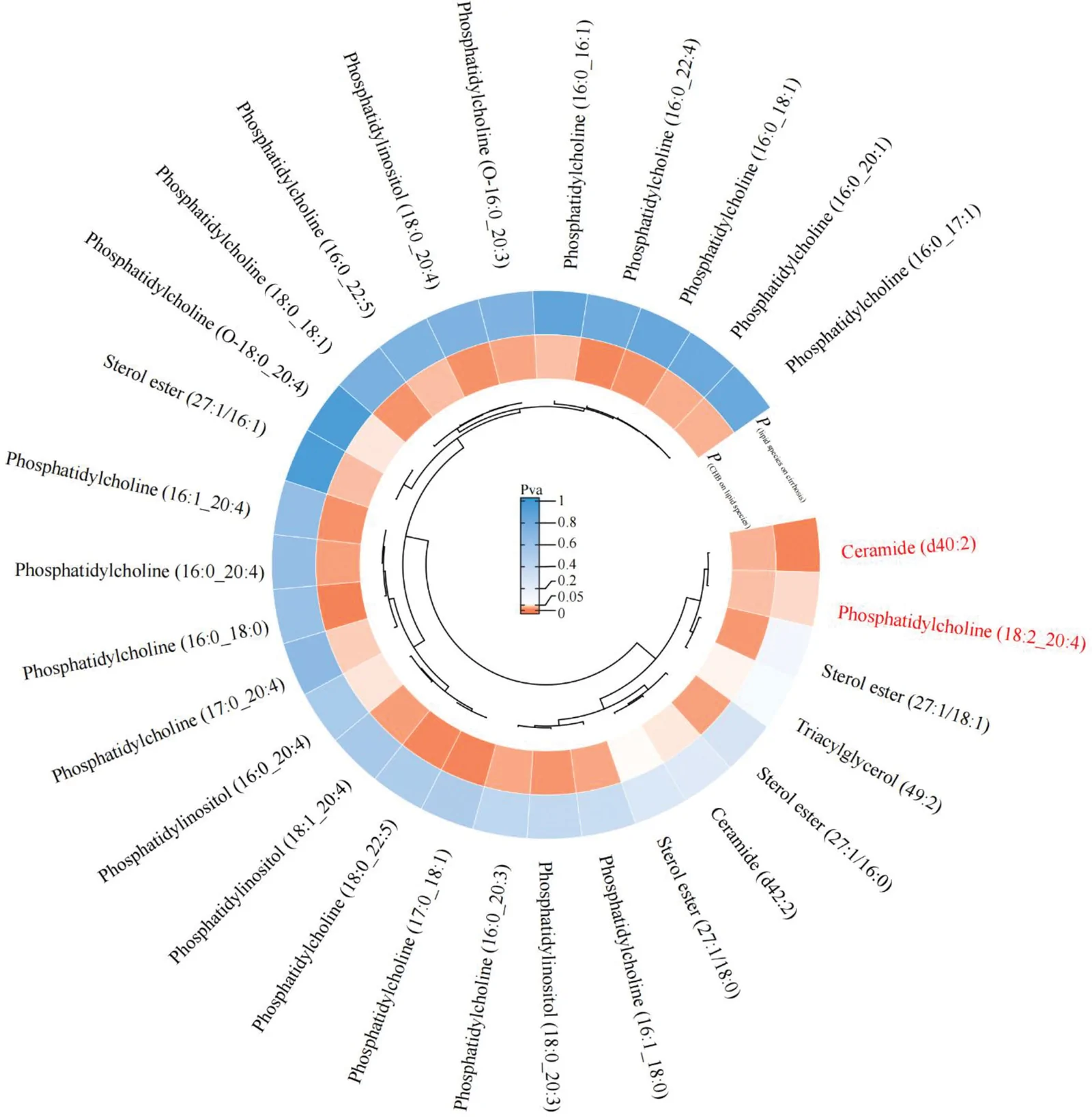
Summary of lipid species associated with CHB and cirrhosis. A circular heatmap illustrating the associations between lipid species and CHB/cirrhosis. The color scale represents the strength of association (*P*-values), with red indicating stronger associations and blue indicating weaker associations. CHB = chronic hepatitis B.

**Figure 5.**
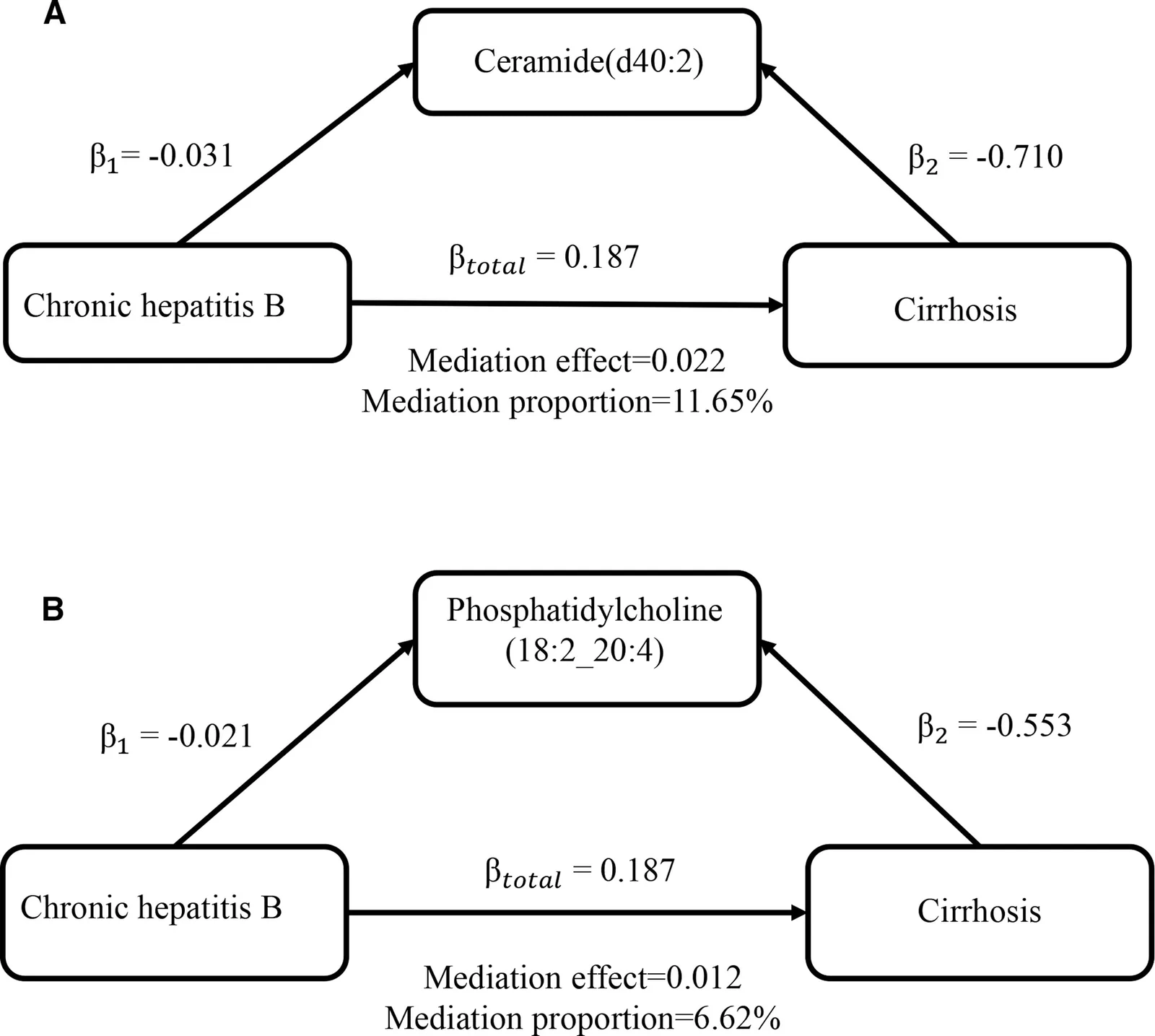
Mediation effect of lipid species in the causal pathway from CHB to cirrhosis. (A) The mediation effect of ceramide (d40:2) in the causal effect of CHB on cirrhosis. (B) The mediation effect of phosphatidylcholine (18:2_20:4) in the causal effect of CHB on cirrhosis. CHB = chronic hepatitis B.

## 4. Discussion

Based on MR analyses, our study suggests that specific lipid species may play a key role in mediating the progression from CHB to cirrhosis. Our MR analysis indicates that ceramide (d40:2) and phosphatidylcholine (18:2_20:4) account for 11.65% and 6.62% of the mediating effect, respectively, in the causal pathway from CHB to cirrhosis. These results suggest that ceramide (d40:2) and phosphatidylcholine (18:2_20:4) may play mediating roles in this process. Even modest mediation effects could be clinically relevant in CHB, and these lipid-mediated pathways may offer both mechanistic insights and potential targets for risk stratification and therapeutic intervention.

Ceramide, a pivotal lipid species messenger at the core of sphingolipid metabolism, plays crucial roles in regulating the physical characteristics of biological membranes, such as facilitating the formation of membrane microdomains. Additionally, it can modulate multiple cell functions.^[37]^ Previous research has indicated that in patients with CHB and cirrhosis, ceramide exhibits a negative correlation with the disease course^[38-40]^; this finding aligns with the effect value outcomes derived from our MR analysis. Moreover, ceramide has the potential to serve as a predictive biomarker for liver function decompensation and may even hold promise as a treatment modality.^[41-43]^ Ceramide and its associated molecules are significantly involved in nearly every step of the viral life cycle.^[44]^ This connection might represent its underlying biological mechanism. Ceramides are known to promote apoptosis,^[45,46]^ and their application is being explored in the context of treatment and prediction.

Phosphatidylcholine is a major component of phospholipids and a crucial constituent of biological membranes. Recent reports have suggested that PC may play a protective role in liver injury.^[47]^ Studies have found that the types and levels of polyunsaturated PC are negatively correlated with liver disease.^[48]^ Phosphatidylcholine has the potential to be used in the treatment of liver diseases, which may be related to its antioxidant effect.^[49]^ Polyene phosphatidylcholine (PPC) is a widely used hepatoprotective drug.^[50]^ The hepatoprotective effect of polyene phosphatidylcholine against CHB has been confirmed.^[51]^ Our findings indicate that phosphatidylcholine (18:2_20:4) may act as a protective factor and mediate the progression from CHB to cirrhosis, further supporting the protective role of PC in HBV-related liver disease.

According to the HBV treatment guidelines, the primary goal of CHB treatment is to inhibit viral replication by using antiviral medications.^[52-55]^ Other treatments and therapeutic approaches are recommended to delay the progression of liver failure, improve the quality of life, and extend life expectancy. Among these, antifibrosis treatment is also an important part of the comprehensive management strategy. It aims to control and halt the progression of liver fibrosis to cirrhosis, thereby reducing the risk of developing cirrhosis and cancer. lipid species, a diverse group of complex-structured biomolecules, is involved in numerous body processes. In the liver, lipid metabolism is crucial.^[7]^ Given its role in physiological balance and potential for treating diseases via lipid-pathway targeting, it could be an attractive target. We conducted the first MR study to explore the causal relationship among CHB, plasma lipid species, and cirrhosis. In this study, the inclusion of large-scale GWAS data enhanced the analytical power. Additionally, we employed various sensitivity analysis techniques to mitigate the influence of confounding factors, thus ensuring the reliability of our causal effect estimates derived from observational studies. Our findings provide a very promising direction for the exploration of using lipid species in the treatment.

There are still some limitations of this study. First, all the populations included in this study are European and are unable to represent the entire population, which may lead to selection bias. This limitation should be considered when interpreting our findings. Future studies should strive to tackle these challenges by adhering to strict methodology and broadening the scope of analysis to incorporate more diverse populations.^[29]^ Future studies with larger cohorts are warranted to confirm the consistency and generalizability of the findings in this study. Second, the lipid species were selected only for analysis in this study; this factor might not capture the entire spectrum of potential biological interactions and effects. A more extensive lipidomic approach can provide a more comprehensive understanding of lipidomic characteristics and their biological impacts. Additionally, the metabolome and microbiome can be incorporated for a more thorough analysis in future studies. Third, our results remain theoretical and await validation through clinical or animal experiments. Further cellular, animal, and clinical investigations are warranted to elucidate these mechanisms.

## 5. Conclusion

This study used the MR analyses to explore the role of the plasma lipid species in the causal relationship between CHB and cirrhosis. The identified lipid species may serve as potential biomarkers for the diagnosis or treatment of cirrhosis. The MR analyses may help researchers to explore the mechanisms that address the progression from CHB to cirrhosis. Future studies that explore early intervention and treatment to prevent cirrhosis are still needed.

## Acknowledgments

We are grateful for resources from the High-Performance Computing Center of Central South University. We thank the European Bioinformatics Institute (EBI) for providing the data publicly and all GWAS participants for their contributions to the summary of statistics data.

## Author contributions

**Conceptualization:** Biao Xiao, Min Zhang.

**Data curation:** Biao Xiao, Qixiang Song, Jie Huang.

**Formal analysis:** Biao Xiao.

**Investigation:** Biao Xiao, Min Zhang.

**Supervision:** Erik De Clercq, Guangdi Li.

**Visualization:** Biao Xiao, Qixiang Song, Zhenggang Wang, Ying Liu, Boqi Li.

**Validation:** Qinghai Zeng, Yelin Deng, Ewelina Krol, Ricardo Khouri.

**Writing – original draft:** Biao Xiao, Min Zhang.

**Writing – review & editing:** Min Zhang, Guangdi Li.

## Supplementary Material


